# Fibroblast clearance of damaged tissue following laser ablation in engineered microtissues

**DOI:** 10.1063/5.0133478

**Published:** 2023-03-14

**Authors:** Megan Griebel, Anish Vasan, Christopher Chen, Jeroen Eyckmans

**Affiliations:** 1 Department of Biomedical Engineering and the Biological Design Center, Boston University, Boston, Massachusetts 02215, USA; 2 Wyss Institute for Biologically Inspired Engineering, Harvard University, Boston, Massachusetts 02115, USA

## Abstract

Although the mechanisms underlying wound healing are largely preserved across wound types, the method of injury can affect the healing process. For example, burn wounds are more likely to undergo hypertrophic scarring than are lacerations, perhaps due to the increased underlying damage that needs to be cleared. This tissue clearance is thought to be mainly managed by immune cells, but it is unclear if fibroblasts contribute to this process. Herein, we utilize a 3D *in vitro* model of stromal wound healing to investigate the differences between two modes of injury: laceration and laser ablation. We demonstrate that laser ablation creates a ring of damaged tissue around the wound that is cleared by fibroblasts prior to wound closure. This process is dependent on ROCK and dynamin activity, suggesting a phagocytic or endocytic process. Transmission electron microscopy of fibroblasts that have entered the wound area reveals large intracellular vacuoles containing fibrillar extracellular matrix. These results demonstrate a new model to study matrix clearance by fibroblasts in a 3D soft tissue. Because aberrant wound healing is thought to be caused by an imbalance between matrix degradation and production, this model, which captures both aspects, will be a valuable addition to the study of wound healing.

## I. INTRODUCTION

Dermal wound healing is a sequential process characterized by hemostasis, inflammation, new tissue formation, and remodeling, four overlapping phases that must occur in a timely manner to restore barrier function and prevent infection. While these phases are considered to be preserved regardless of the type and location of the wound,^1^ the duration and clinical outcome of healing depend on the cause of injury, type and size of the wound, and contamination state. As such, different wound care strategies to achieve the optimal outcome have been developed.^2,3^ Yet, the mechanisms by which injury modality affect the healing process remain poorly understood.

Despite the pervasive clinical presentation of various types of wounds, such as ulcers, abrasions, lacerations, and burns, there are few studies comparing tissue damage and the wound healing response between different injury modalities. Early animal studies comparing burn wounds to excision wounds show that burns heal more slowly than skin excision wounds^4^ and suggest that the proliferation phase of healing is delayed in burns compared to size-controlled cuts.^5^ Unlike cuts and lacerations, burns do not exhibit hemostasis^2^ and also have substantially more damaged tissue to be cleared, both of which may contribute to the slower healing process of burns. Comparisons have also been made in a zebrafish injury model: delayed healing of thermal injuries compared to transection injuries was attributed to a disruption in the structure of collagen fibers.^6^ Close examination of the extracellular matrix (ECM) in skin tissue in multiple animal models showed that thermal injuries created a region of denatured collagen detected by second harmonic generation (SHG) imaging,^7^ loss of birefringence,^8^ and a Cy5 labeled collagen-mimetic peptide that specifically binds denatured collagen.^9^ These studies suggest that injury modalities can cause different degrees of damage to surrounding tissue, which may affect the progression of the healing stages.

During the inflammatory phase, necrotic tissue, including dead cells and damaged ECM, and pathogens are removed from the wound bed,^10^ a process hereinafter referred to as wound clearance. Wound clearance has been mainly attributed to immune cells, particularly neutrophils and macrophages that engulf bacteria and apoptotic cells via a process called phagocytosis. When phagocytic clearance of apoptotic cells by macrophages is impaired, wound healing is delayed.^11,12^ Interestingly, phagocytic behavior is not limited to immune cells; other cell types, including epithelial cells and fibroblasts, can clear apoptotic cells through phagocytosis.^13–15^ In addition to clearance of apoptotic cells, damaged ECM must also be removed from the wound bed. Both macrophages and fibroblasts secrete proteolytic enzymes such as matrix metalloproteinases (MMPs) that cleave ECM molecules.^16^ However, how the degraded matrix leaves the wound scene is less clear. In healthy tissues, fibroblasts engulf collagen and other ECM proteins to regulate collagen maintenance and turnover.^17,18^ Whether fibroblasts actively contribute to the clearance of damaged ECM following tissue injury remains elusive.

To begin to address this question, we set out to study tissue repair in the presence of varying amounts of damaged tissue in our previously reported biomimetic model for stromal wound healing.^19^ This model uses 3T3 fibroblasts that compact a collagen type I hydrogel around elastomer pillars to form a fibrous microtissue. Upon injury with a microdissection knife, fibroblasts migrate into the gap, smoothen the wound edge, and subsequently repair the gap through assembly of a fibronectin-rich provisional matrix.^19^ In this study, we selected laser ablation as an alternate mode of injury because of its amenability to create wounds at the desired scale (on the order of 100–400 *μ*m) without dislodging the suspended microtissue. We first demonstrate that transection and laser ablation cause different degrees of tissue damage, which results in altered wound healing dynamics. While healing occurred in both wound types, laser ablated wounds exhibited a prolonged opening phase after injury compared to transected wounds, wherein fibroblasts invade and remodel the damaged tissue surrounding the wound. Closer examination with transmission electron microscopy revealed that fibroblasts engulfed damaged ECM from laser ablated wounds through a process that was mediated by RhoA and dynamin activity. These data suggest a previously unappreciated role for fibroblasts in wound clearance.

## II. RESULTS

### A. Laser ablation creates holes in microtissues surrounded by non-viable tissue

**FIG. 1.**
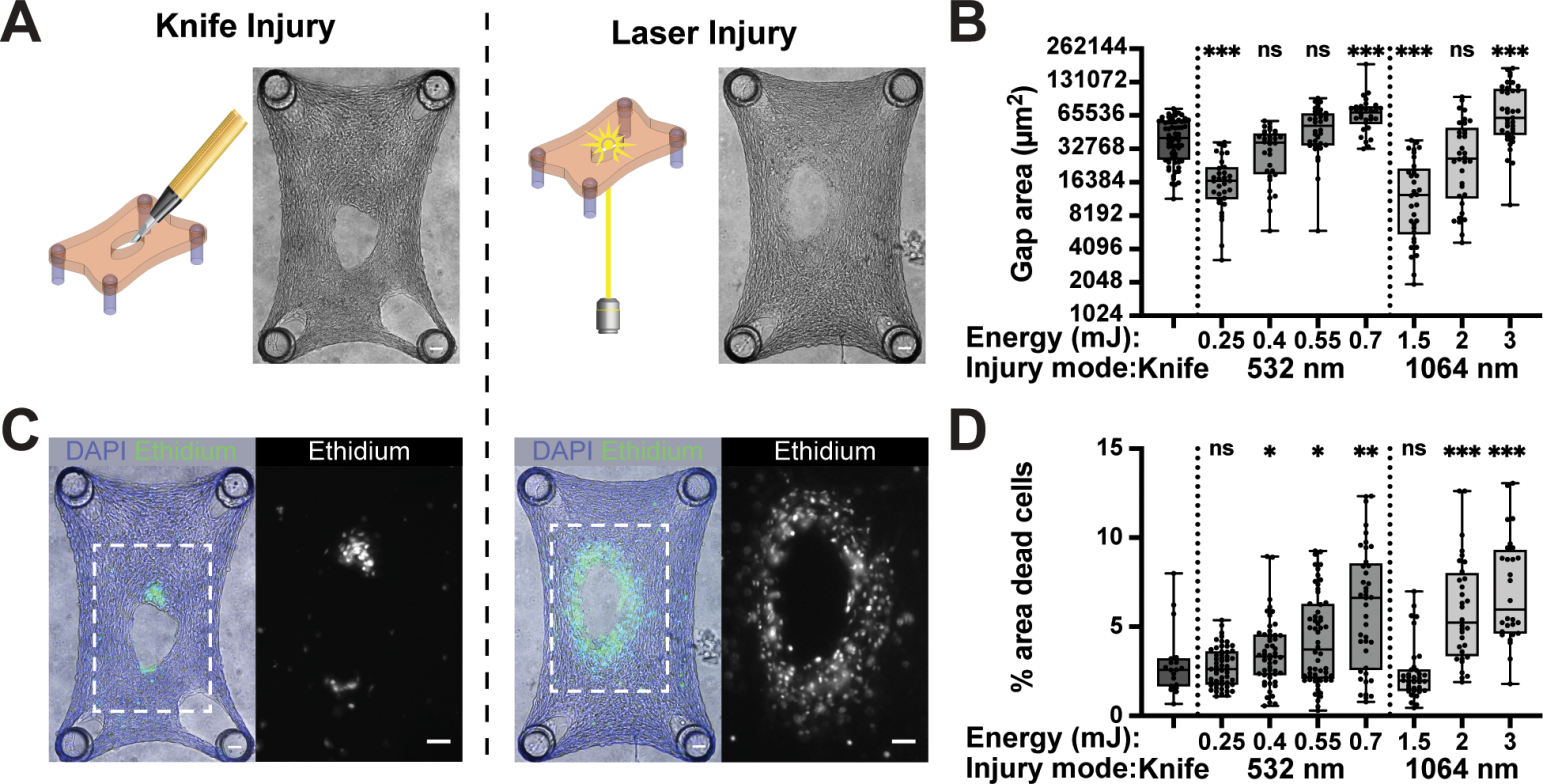
Tissue injury by transection and laser ablation in stromal microtissues. (a) Two modes of injury are used to generate full-thickness gaps in microtissues. Phase contrast images show tissues immediately following injury. Scale bars: 100 *μ*m. (Left) A diamond microdissection knife was used to manually create gaps in tissues. (Right) An Nd:YAG laser pulse, at 532 or 1064 nm, focused through a 10× objective, ablates a gap in the tissues. (b) Quantification of initial gap area following injury. Laser gap sizes are shown for distinct energy levels. (c) Epifluorescence images showing Hoechst stain for all cell nuclei (blue) and ethidium homodimer-1 stain for nuclei of dead cells (green/white). Scale bars: 100 *μ*m. (d) Quantification of percentage of tissue area covered by dead cells [from (c), white area/blue area]. Data in panels (b) and (d) were compared to the knife injury condition using the non-parametric steel test with control. * p < 0.05, ** p < 0.01, *** p < 0.001, and ns: not significant.

In our previous studies, stromal microtissues were injured using a microdissection knife mounted to an xyz-micromanipulator.^19–21^ Due to the soft and compliant properties of microtissues, mechanical injury often resulted in slipping of the microtissue from the pillars before a gap was created. To overcome this limitation, we sought to deploy a Q-switched Nd:YAG nanosecond-pulsed laser to inflict wounds with laser ablation [Fig. 1(a)]. The Nd:YAG laser operates at 1064 nm, and our setup includes a removable KTP crystal capable of generating the second harmonic at 532 nm. Each pulse width was 5 ns. Given the different mode of injury, transection vs ablation, we set out to characterize the two wound types and performed a viability assay on microtissues injured with either a microdissection knife or laser pulse. Interestingly, we observed a key difference in cell viability at the wound periphery. In knife-injured tissues, the long edges of the wound were largely free of dead cells as if the dead tissue was almost entirely removed, leaving only a small patch of dead cells at the end of the incision [Fig. 1(c)]. In contrast, laser-injured microtissues showed a band of dead cells around the perimeter of the wound [Fig. 1(c)] indicating that tissue damage extended beyond the wound margins into the surrounding tissue.

To establish a protocol for reproducibly inflicting full-thickness gaps with laser light, microtissues were injured with laser pulses of different wavelengths (532 or 1064 nm) and pulse energies ranging from 0.25 to 3 mJ, two parameters that affect the energy density of the focused laser pulse. Phase contrast images were captured following injury [Fig. 1(a)], and the initial gap area was quantified [Fig. 1(b)]. At both wavelengths, 532 and 1064 nm, there was a threshold energy below which no tissue gap was created, which was about 0.25 mJ at 532 nm and 1.5 mJ at 1064 nm. These laser pulse energies correspond to a fluence, which is the energy density at the focal point in the tissue, of 19 and 17.6 kJ/cm^2^ for 532 and 1064 nm, respectively (supplementary material, Table S1). Thus, the ablation threshold of collagenous microtissues was similar for both wavelengths. Because laser pulse energies were measured before each experiment, we reported laser pulse energies, and not estimated fluences, in the x-axis of the graphs. At the minimum energies, the gaps created were smaller (mean values of 17 600 ± 1540 *μ*m^2^ at 532 nm and 14 300 ± 3000 *μ*m^2^ at 1064 nm) than those created by knife (41 600 ± 3980 *μ*m^2^). At intermediate energies, 0.4 and 0.55 mJ at 532 nm and 2 mJ at 1064 nm, the average gap size (34 200 ± 4270 *μ*m^2^; 53 800 ± 8500 *μ*m^2^; and 30 100 ± 6670 *μ*m^2^, respectively) was on the same scale as knife injuries. Further increase in the pulse energy for both wavelengths yielded initial gap areas that were much larger than what the knife was capable of creating [Fig. 1(b)]. To assess damage to the tissue surrounding the gap area, knife and laser injured microtissues were stained with ethidium homodimer-1 to detect dead cells with damaged cell membranes, and the percentage of area that contained dead cells was quantified for different wavelengths and pulse energies [Figs. 1(c) and 1(d)]. The area of non-viable tissue in all but the lowest energy injuries was significantly larger than in the knife injuries, although there was a large range in size of the damaged zone [Fig. 1(d)]. Together, these data show that laser ablation can be used to reliably create gaps in microtissues with sizes comparable to knife inflicted wounds, but with more cell damage in the adjacent tissue.

### B. Ablated microtissues close following an initial opening phase

**FIG. 2.**
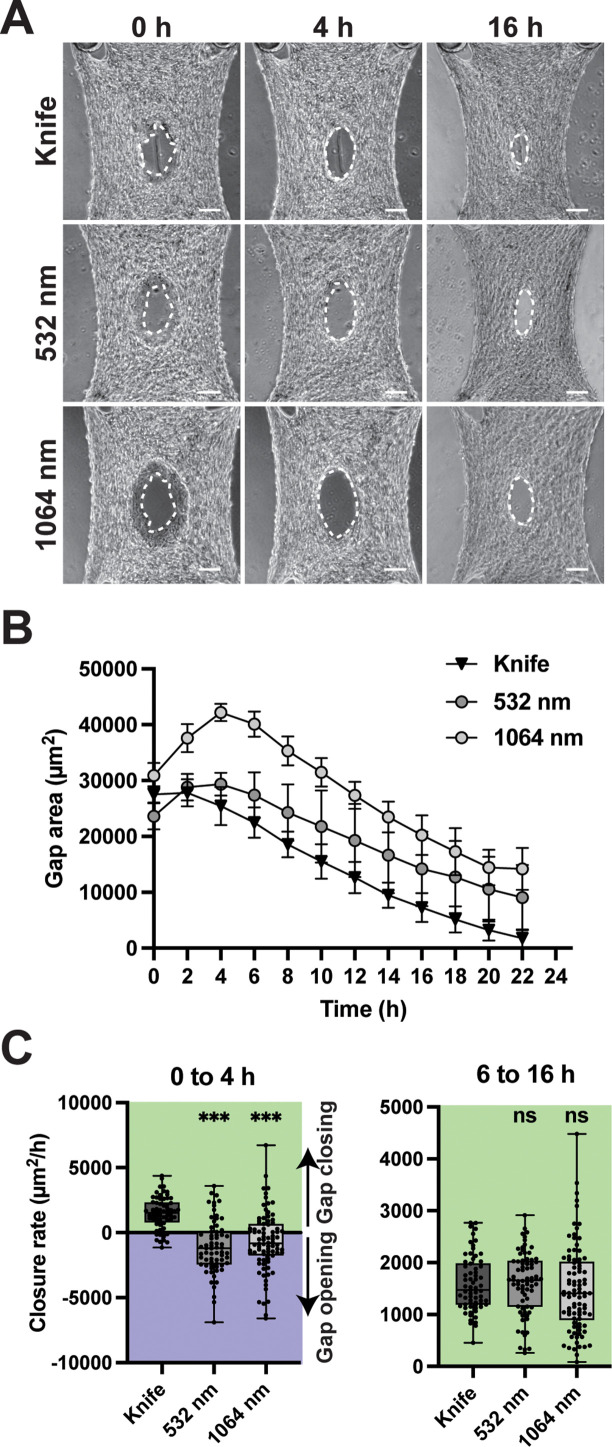
Closure of injured microtissues. (a) Time-lapse images are shown at 0 , 4, and 16 h for each injury mode. The white dotted line indicates gap edges. Scale bars: 100 *μ*m. (b) Gap area as a function of time for each injury mode. Mean and standard deviation for n = 4 tissues per mode with comparable starting areas. (c) Closure rate for the initial phase during the first 4 h of the time-lapse (left) and between 6 and 16 h (right). Data are compared to knife condition using Dunnett's test with control. *** p < 0.001 and ns: not significant. Multimedia view: https://doi.org/10.1063/5.0133478.1
10.1063/5.0133478.1

Gap closure in our model system is initiated by the fibroblasts adjacent to the wound edge.^22^ Given the increased levels of cell death in laser-ablated wounds vs knife wounds [Figs. 1(c) and 1(d)], we hypothesized that ablation wounds would show impaired closure. To investigate this hypothesis, we monitored gap closure for stromal microtissues injured by knife and by laser, both 532 and 1064 nm, using time-lapse microscopy. As previously reported, knife-injured tissues smoothed the wound margins and commenced closure within minutes, and closure finished in about 24 h^19^ [Fig. 2(a), Multimedia view]. The laser-ablated tissues displayed a prolonged smoothing phase that was characterized by opening of the gap that lasted between 4 and 6 h, which was then followed by closure similar to knife-injured tissues [Fig. 2(b)].

To quantitatively assess tissue closure between the different conditions, we compared closure rates during two phases of the healing process. We defined the initial phase between 0 and 4 h because ∼80% of tissue gaps reached their maximum area by 4 h. We considered the closure phase to occur between 6 and 16 h post injury as most tissue gaps (>90%) reached the maximum area by 6 h, and the closure rate was linear for the ensuing 10 h [Fig. 2(b)]. The rate of closure during each phase was quantified as change in gap area per hour, where a negative value indicates the gap was opening rather than closing [Fig. 2(c)]. During the initial phase [Fig. 2(c) left], knife injured tissues displayed an average closure rate of 1380 ± 270 *μ*m^2^/h. For 532 nm injuries, the closure rate during this phase was −890 ± 820 *μ*m^2^/h (p < 0.001 compared to knife). For 1064 nm injuries, the closure rate during this first phase was −870 ± 390 *μ*m^2^/hour (p < 0.001 compared to knife). During the closure phase, the closure rates were not significantly different between injury modes [Fig. 2(c) right]. Together, these data suggest that a prolonged opening phase, rather than an impaired closure phase, delayed gap closure in laser ablated wounds.

### C. Characterization of fiber damage and movement at gap periphery

To investigate the gap opening in laser inflicted wounds, we carefully analyzed the time lapse phase contrast images during the opening phase and observed a darker band of damaged tissue at the periphery of the gap [Fig. 2(a)] that corresponded to the area of dead cells [Figs. 1(c) and S2). In phase contrast microscopy, changes in apparent brightness are due to phase shifts in light that occur when light passes through the sample. Therefore, visually darker tissue indicates that a different phase shift occurred in these parts of the tissue, potentially due to altered topography or density (increased or decreased density relative to the bulk tissue could both cause this visual distinction). This band of damaged tissue was present in most ablated tissues; very few knife injured tissues contained a small patch of damaged tissue, but not a circumferential band (Fig. S2). During the initial opening phase, this band grew smaller [Fig. 2(a)] as the gap area increased [Fig. 2(a) white dotted lines, Fig. 2(b)]. We did not observe significant differences between the two laser wavelengths in the clearance and repair process. Although not statistically significant, the laser seems to generate more peripheral tissue damage at 1064 nm. For these reasons, and to simplify experiments, the laser was used in 1064 nm mode from this point onward in the study.

**FIG. 3.**
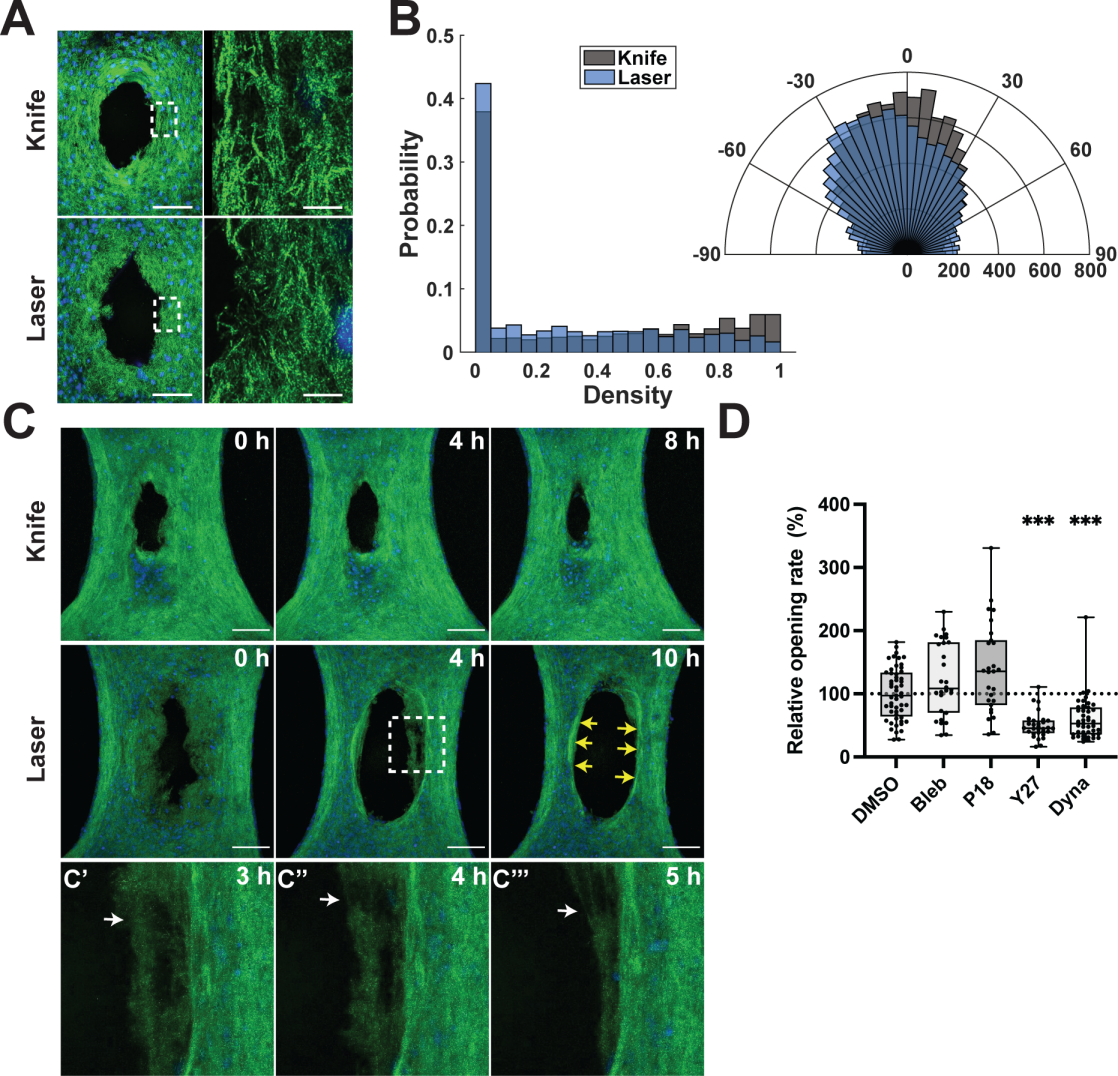
Fiber disruption and breakage following ablation. (a) Hoechst dye (blue) and reflected 488 nm illumination (green). (Left) Max projection of tissue z-stacks. Scale bars: 100 *μ*m. White box correlates with region shown on right. (Right) Single z-slices of 75× images. Scale bars: 10 *μ*m. (b) Histogram in polar coordinates showing frequency (r-axis) of net fiber orientations ( θ-axis) of 10 pixel2 subregions for 25 *μ*m of tissue adjacent to the gap long edge. Histograms are significantly different according to Kolmogorov–Smirnov test, p < 0.001. (c) Max z-projections of time-lapse reflection microscopy. Yellow arrows show compacted tissue at gap edge at the conclusion of the clearance phase. Scale bars: 200 *μ*m. (c′)−c‴) Enlargement of area inside white box from (c), laser injured tissue, showing fiber breakage at 3 h (c′), 4 h (c″), and 5 h (c‴). White arrows indicate breakage site. (d) Opening rate, normalized to the mean control (DMSO) rate within each biological replicate, during the first 4 h following ablation. Non-parametric steel test with control. *** p < 0.001. Multimedia view: https://doi.org/10.1063/5.0133478.2
10.1063/5.0133478.2

From the time-lapse images in Fig. 2, we postulated that the ECM at the gap periphery was compromised and, therefore, cleared by the remaining fibroblasts in the microtissue. In order to characterize the mechanical damage to surrounding ECM caused by each type of injury, we sought to visualize the microstructure of the fibrous matrix. We used high-resolution, reflection microscopy to capture z-stack images of the matrix topography at the wound edges. From these images, we made two main observations. First, the depth of the tissues in z was significantly smaller for knife (110 ± 14 *μ*m) than for laser (152 ± 18 *μ*m) injuries (p < 0.001). Qualitatively, the knife-injured tissues appear to have a higher density of fibers than do ablated tissues [Fig. 3(a)], suggesting that the mechanical injury compacts the microtissue, likely due to the pressure from the microdissection knife. To quantify fiber density, the 25 *μ*m region closest to the gap edge was divided into subregions and the density of each calculated. The histogram showing fiber density within these subregions reveals that laser injuries contain more empty space than do knife injuries [Fig. 3(b), left, p < 0.001]. Second, the fiber structure at the gap edge appeared disrupted in ablated tissues. We used an established algorithm^23^ based on the Fibril Tool method^24^ to determine the distribution of fiber orientation angles within 25 *μ*m of the gap edge. This analysis revealed that the majority of fibers in knife-injured tissues aligned along the long axis of the tissue, parallel to the direction of movement of the knife. The peak alignment angle shifts to about −20°, and a wider spread of orientation angles was observed for ablation injuries compared to mechanical injuries [Fig. 3(b), right, p < 0.001]. Together, these data suggest that tissue gaps made with laser ablation are surrounded by damaged tissue that is composed of dead cells and compromised ECM.

We wondered next how the fibrous matrix structure changes as the fibroblasts remodel the microtissue at the gap edge. Using reflection microscopy, we performed live microscopy and captured images every 10 min [Fig. 3(c), Multimedia view]. In mechanical injuries, the gap edge smooths and begins closure within 4 h. For ablation injuries, we observed the tissue from the damaged gap periphery moving into the bulk tissue over a period of 4–10 h. In order to maximize the amount of damaged tissue that we could observe, wounds in this experiment only were made roughly three times larger than those in Fig. 2. Therefore, they take roughly three times longer to complete the clearance phase and reach their maximum wound size. In some places, fiber breakages occurred as the damaged matrix was remodeled [Figs. 3(c′) and 3(c‴), white arrows]. At the conclusion of this tissue clearance phase, we observed compacted tissue at the wound edges [Fig. 3(c), yellow arrows]. In order to confirm that the fibrous tissue remodeling was due to cell activity, rather than a passive feature of the collagenous hydrogel, we treated the microtissues with a potent inhibitor of actin polymerization, latrunculin A, which caused the cells in the microtissue to round up and prevented all remodeling of the wound edges (Fig. S3).

Given the fiber breakages and compaction of the damaged matrix, we postulated that actomyosin contractile forces were required for tissue clearance [Fig. 3(d)]. The data in Fig. 3(d) are reported as relative opening rate for each treatment compared to the vehicle control (DMSO). Laser ablation was used for all conditions; because we sought to observe changes in damaged tissue clearance, and knife injuries did not display this clearance phase, the knife condition was not included in these experiments. To decrease cellular contractility, the myosin-2 inhibitor blebbistatin (100 *μ*M), myosin light chain kinase (MLCK) inhibitor peptide 18 (P18, 10 *μ*M) or Y27632 (10 *μ*M), and a Rho-associated kinase (ROCK) inhibitor were added prior to microtissue ablation. Interestingly, blebbistatin and P18 did not significantly affect the tissue opening rate, while Y27632 significantly decreased the mean tissue opening rate to 50% ± 1% of the vehicle control, DMSO (p < 0.001). These results suggest that ROCK activity, but not cellular contractility, is required for wound clearance in our system. In addition to modulating cellular contractility, ROCK II activity is also an important regulator of phagocytosis of FN-coated beads in fibroblasts.^25^ To investigate if fibroblasts cleared the wound by engulfing damaged matrix, we added dynasore (60 *μ*M) to inhibit dynamin activity, which is necessary for phagocytosis^26,27^ and clathrin-mediated endocytosis.^27^ Indeed, addition of dynasore lowered the mean opening rate to 57% ± 9% of control (p < 0.001), suggesting that ECM ingestion may play a role in damaged tissue clearance by fibroblasts.

### D. Transmission electron microscopy reveals intracellular fibrillar structures, suggesting phagocytosis of ECM after ablation

**FIG. 4.**
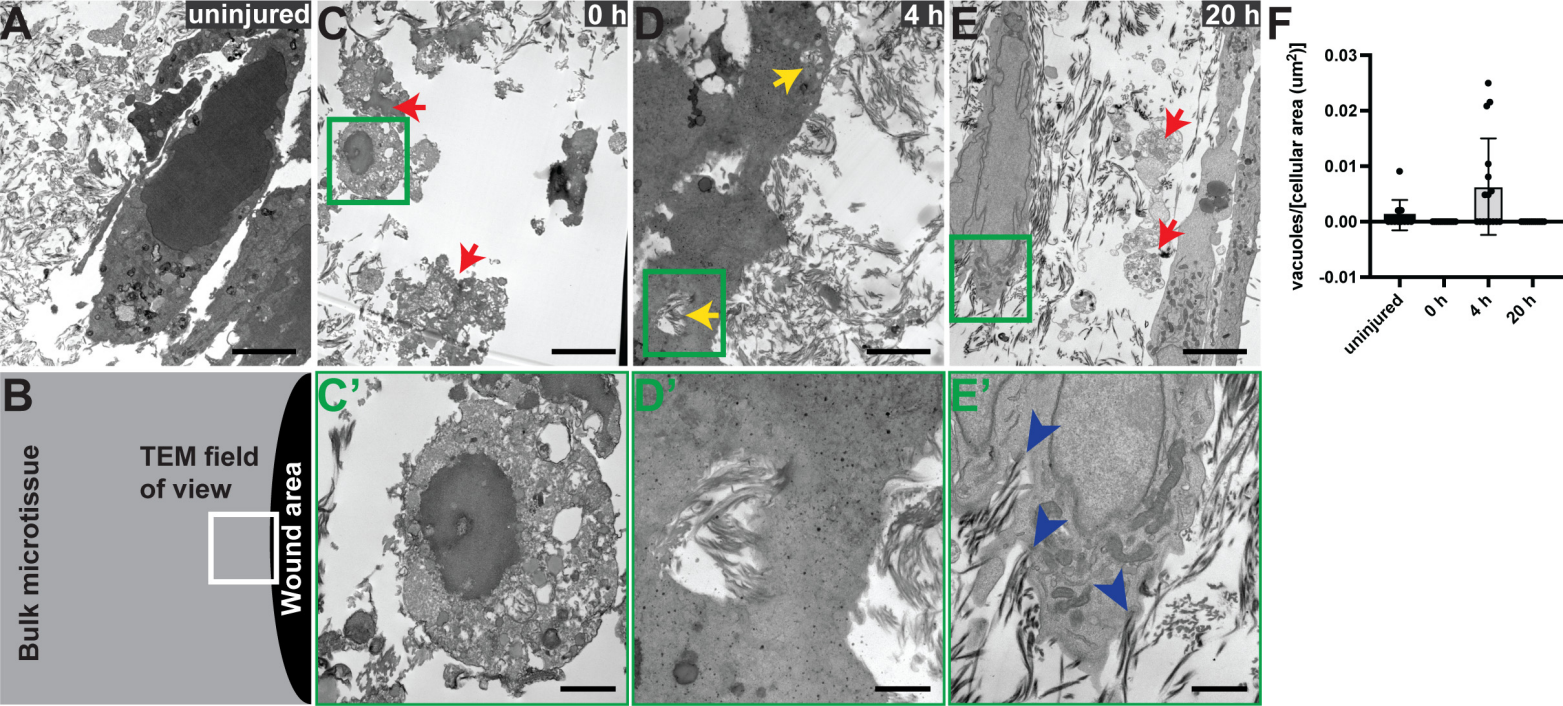
Transmission electron microscopy suggests ECM fiber endocytosis following ablation. (a) Uninjured microtissue. (b) Schematic of where the field of view was located in the microtissues for (c)−(e). (c) Fibroblasts at gap edge fixed immediately after ablation. Red arrows indicate dying cells, characterized by reduction in cell size, fragmented nuclei, many vacuoles, and blebbing. (d) Cells at gap edge 4 h after injury. Yellow arrows indicate vacuoles containing ECM fibers. (e) Cells at gap edge 20 h after ablation. Red arrows indicate non-viable cells. The cell on the left has an enlarged nucleus and many mitochondria. (a)−(e) Scale bars: 4 *μ*m. (F) Quantification of number of vacuoles containing distinct fibrillar structures normalized to cellular area for uninjured tissue and tissue 0, 4, or 20 h after ablation. n = 10 fields of view. (c′) Magnified view of the green box from (c), immediately following ablation. An oncotic or necrotic cell, characterized by reduction in cell size, chromatin condensation at nucleus edge, and the formation of many cytoplasmic vacuoles. (d′) Magnified view of the green box from D, 4 h after ablation. An intracellular vacuole containing fibrillar structures matching the distinct appearance of the extracellular matrix. (e′) Magnified view of the green box from (e), 20 h after ablation. Blue arrowheads indicate extracellular matrix fibers that appear to be emanating from the cell. (c′')–(e′) Scale bars: 1 *μ*m.

To further corroborate the hypothesis that fibroblasts clear wounds by engulfing ECM, we performed transmission electron microscopy (TEM) on uninjured microtissues and at the wound edge of laser injured microtissues at 0, 4, and 20 h post injury (Fig. 4). Immediately following ablation, cells at the wound edge displayed fragmented or misshapen nuclei and showed cellular blebbing [Figs. 4(c) and 4(c′), red arrows]. Other cells showed the formation of numerous, apparently vacant, cytoplasmic vacuoles, a reduction in cell size, and chromatin condensation at the edge of the nucleus [Fig. 4(c′)], which is reminiscent of a necrotic or oncotic cell.^28,29^ At 4 h after ablation, cells at the wound edge contained vacuoles with distinct fibrillar structures [Fig. 4(d), yellow arrows and Fig. 4(d′)]. These vacuoles were relatively large (>1 *μ*m in diameter), and the fibrillar structures appeared to have a similar morphology to those of the surrounding damaged extracellular matrix. At 20 h after ablation, cells at the wound edge lacked vacuoles, but instead contained numerous organelles, including mitochondria [Fig. 4(e)]. This observation suggests increased metabolic activity, as would be expected of fibroblasts actively migrating and producing new ECM, two ATP intensive processes. Close inspection of the cell surfaces near the wound edge revealed that fibrillar structures appear to be emanating from the cell [Fig. 4(e′), blue arrowheads], which is in line with our previous studies showing that fibroblasts in microtissues close wounds by producing a provisional matrix that is rich in fibronectin and collagen type III.^19–21^ Remnants of necrotic cells can still be seen at 20 h after injury [Fig. 4(e), red arrowheads]. However, following closure, the number of necrotic cells was drastically reduced compared to one hour after injury (Fig. S4), suggesting that dead cell debris may also be cleared by fibroblasts. Although this observation is in line with a recent study showing that dermal fibroblasts phagocytose apoptotic cells,^30,31^ it remains to be seen if 3T3 fibroblasts phagocytose dead cell debris in collagenous microtissues.

In order to determine whether ECM phagocytosis occurred at all times in the microtissues, or whether it was an injury-specific response, we quantified the number of vacuoles that contained distinct fibrillar structures [as in Fig. 4(d′)] for 10 fields of view at each timepoint. In uninjured tissues, 6 such vacuoles were observed, and in tissues 4 h post injury, 17 such vacuoles were observed. Only vacant vacuoles were seen immediately following injury, and thus, not included in this quantification. No vacuoles were observed at 20 h post injury. Due to differences in number of cells and cell sizes, the number of fiber-containing vacuoles was normalized to cellular area [Fig. 4(f)].

## III. DISCUSSION

In this study, we explored laser ablation as a method to create full thickness wounds in engineered stromal microtissues and found that fibroblasts first clear the wound from damaged ECM before a new provisional matrix is assembled to close the wound. Previous studies have established a critical role for phagocytic macrophages^11,12^ and non-professional phagocytic epithelial cells^13–15^ to participate in the removal of cell debris and apoptotic cells, but it is unclear if these cells actively remove damaged ECM from the wound site. In our experiments, dead cells remained present during the healing of microtissues, but the ECM at the site of injury was engulfed by fibroblasts via a process that was dependent on ROCK and dynamin activity. These data are in line with other studies showing that, in non-wound healing settings, fibrillar collagen can be digested by cells' integrin-dependent phagocytosis,^17,18,32^ nonspecific macropinocytosis,^18,33^ and Endo180/uPARAP-mediated endocytosis.^18,34^ While further studies need to be conducted to delineate the mechanisms by which damaged collagen fibers are removed from the injury site in our model system, our data highlight a new role for fibroblasts in wound clearance.

*In vitro* wound healing models have been instrumental tools to parse out mechanisms of cell migration,^35^ vascularization,^36^ and tissue assembly^19,37,38^ during wound healing. In these model systems, wounds are often generated by removing cells or tissue with a scalpel, pipet tip, or biopsy punch leaving a clean wound with limited cell death or devitalized tissue present in the wound bed. Similar to what others have reported in these clean wounds, in this study, microtissues injured with a microdissection knife showed limited cell death or tissue damage, resulting in the immediate onset of tissue closure. In contrast, microtissues subjected to laser ablation showed open wounds surrounded by a zone of damaged ECM that contained elevated numbers of dead cells. This wound morphology is likely caused by a fast microscale thermal explosion in the focal point of the laser pulse that removes tissue in the center of the microtissue with the ensuing heat dissipation denaturing the surrounding tissue.^39,40^ Hence, laser ablated wounds show similarities to thermal injuries such as burns.

Analogous to the healing dynamics of burn wounds in animals,^4,5^ the presence of devitalized tissue in our model delayed the onset of wound closure. During this period of delay, fibroblasts were found to be actively clearing the wound of denatured ECM prior to assembling a provisional matrix to close the gap. The coordination between ECM clearance and deposition of new matrix is critical for normal healing as imbalances between these two processes are implicated in pathological healing such as fibrosis^41,42^ and chronic healing.^43^ In these settings, the surgeon removes the compromised wound bed by performing a surgical or enzymatic debridement in order for healing to proceed.^44^ Understanding how to increase natural (autolytic) debridement or identifying mechanisms underlying regulation of ECM clearance could prove useful in treating chronic and fibrotic wounds. Bioengineered models of wound healing that capture both processes, ECM clearance and assembly, could be powerful *in vitro* platforms to gain such insight and to identify new therapeutic targets for chronic healing and fibrotic diseases.

## IV. METHODS

### A. PDMS device fabrication

As previously described,^19,45^ standard photolithography was used to create an SU8 model of the microtissue device. A negative mold of the device was cast using poly-dimethyl siloxane (PDMS, Sylgard 184, Dow-Corning). Finally, the negative mold was used to generate devices of PDMS in 35 mm culture dishes. One device consisted of 80 rectangular microwells, each containing four cylindrical pillars with an overhanging cap near the top to constrain microtissues. Microwells were 1.12 × 1.5 mm^2^, and pillars had 0.62 and 1.0 mm between pillar centers. Pillar diameter was 120 *μ*m for the first layer with a height of 110 *μ*m, and cap diameter was 180 *μ*m with a height of 80 *μ*m.

### B. Cell culture and microtissue seeding

3T3 cells (ATCC #CCL-92) were grown in Dulbecco's modified eagle medium (DMEM, Fisher Scientific #10-013-CV) with 10% bovine (Sigma #B9433) or calf serum (ATCC #30-2030) and 1% penicillin/streptomycin (Life Technologies #15140-122). Cells were mixed with rat tail tendon collagen I (Corning #356236) at a final concentration of 2.2 mg/ml (pH ∼ 7.5). Then, 900 K cells were added to each device and centrifuged to drive cells into the microwells. Excess collagen solution was removed, and collagen in the microwells was allowed to polymerize at room temperature for 10 min, then at 37 °C for 10 min. Microtissues were cultured at 3 °C with 5% CO_2_ in growth medium for 16–20 h prior to injury, during which time they spontaneously compact, as previously described.^19^ Compacted tissues suspended between the PDMS pillars contain about 2000 cells per tissue.^19^

### C. Knife injury and laser ablation of microtissues

For knife injury, a diamond dissection knife (type MDL, Electron Microscopy Systems, #72029) mounted to an XYZ-micromanipulator (SLC-2040, SmarAct GmbH) was used to tear a full-thickness wound in the microtissues [Fig. 1(a)]. Laser ablation was performed using a q-switched nanosecond-pulsed Nd:YAG laser (Minilite I, Continuum), which emits at 1064 nm. For experiments shown in Figs. 1 and 2, the beam was first passed through a Potassium Titanyl Phosphate (KTP) crystal to generate the second harmonic at 532 nm. The beam is enlarged by a beam expander (Edmund Optics #39-739) from 3 to 9 mm, directed through a dichroic mirror suitable for 1064 or 532 nm, and focused into the tissue through a 10× objective. Laser energy was measured directly after the beam expander and was adjusted between 0.25 and 0.7 mJ for 532 nm and between 1.5 and 3 mJ for 1064 nm. Phase images with an Axiovert inverted microscope (Zeiss) at 10× equipped with a 20Mp Blackfly camera (FLIR) were taken immediately following injury to quantify gap starting area. Hoechst 33342 (Sigma #14533) and ethidium homodimer-1 (Thermo Fisher #E1169) were added to each device for 30 min, and epifluorescence images of tissues were captured at 10× with a Nikon TE-200 inverted microscope equipped with a color digital camera. Cell death was quantified as the fraction of dead cell area over total tissue area.

### D. Live microscopy and closure rate measurements

For closure experiments, time-lapse images were taken every 30 min for 18 h on a Nikon Ti Eclipse microscope fitted with an environmental chamber (37 °C, 5% CO_2_). Gap area was measured every hour in ImageJ, manually, using the polygon selection tool. The closure rate was determined as the slope of the line of best fit through data from 0 to 4 h and from 0 to 16 h.

### E. Reflection microscopy and fiber alignment analysis

Reflection microscopy was performed at 488 nm using a 25× water immersion objective with 0.75× digital zoom on an upright Leica SP8 confocal microscope. Samples were excited at 488 nm, and reflected light was captured between 484 and 492 nm. For fiber alignment experiments, tissues were fixed in 4% paraformaldehyde, and Hoechst 33342 was added to visualize cell nuclei prior to imaging. For reflection time-lapse experiments, the microscope chamber was maintained at 37 °C. Tissues were maintained in CO_2_-independent L15 Leibovitz's media. A layer of mineral oil was added atop the growth media to prevent evaporation. Z-stacks of about 30 slices with a z-step size of 4 *μ*m were captured every 10 min for 4 to 8 h.

The fiber alignment analysis was performed in MATLAB (MathWorks, MA) using a previously reported MATLAB code that was based on the Fibril Tool.^23,24^ First, each tissue stack was divided into six equal sections, and the max projection for each section was taken. Second, the rectangular region to be analyzed was defined for each image as the height of the field of view (148 *μ*m) parallel to the tissue long axis, and a width of 25 *μ*m adjacent to the gap edge. Next, a Gaussian blur was applied to images to reduce pixelation effects, then the local pixel intensity gradient was used to determine a perpendicular normalized vector, which was then used to calculate the local nematic tensor for each pixel. The region was then subdivided into 10-pixel × 10-pixel subregions and the average nematic tensor for each subregion was calculated. The largest eigenvector corresponding to the largest eigenvalue of this average describes the alignment direction. Data are reported as a histogram of subregion alignment angles for all 25 × 148 *μ*m regions. For fiber density, an automatic threshold was applied in ImageJ, and the pixel density for the same 10 pixels × 10 pixels subregions was calculated. Data are reported as a histogram of subregion signal density for all 25 × 148 *μ*m regions.

### F. Inhibitor experiments

Inhibitors were added in fresh growth medium 30 min prior to injury. DMSO (Fisher Scientific #BP231) was added at 0.2% (v/v) as a vehicle control. Blebbistatin (Cayman Chemical #24171) was used at 100 *μ*M. Myosin light chain kinase inhibitory peptide 18 (P18, Cayman Chemical #19181) was used at 10 *μ*M. ROCK inhibitor Y27632 (Cayman Chemical #10005583) was used at 10 *μ*M. Nocodazole (Cell Signaling #2190s) was used at 100 nM. Dynasore (Sigma #D7693) was used at 60 *μ*M. Latrunculin A (Sigma #428026) was used at 1 *μ*M.

### G. Transmission electron microscopy

For transmission electron microscopy (TEM), samples were fixed at specified time-points in 2% PFA/2.5% glutaraldehyde overnight. The samples were washed thrice in 0.1 M phosphate buffer (PB) and a secondary (post) fixation was performed in a 1.5% potassium ferricyanide:1% osmium in 0.1M PB in a Pelco^®^ BioWave (Ted Pella, Inc.) at 100 W for 4 min. A subsequent incubation in 1% osmium in 0.1M PB in the BioWave was performed, and samples were then subject to a gradual ethanol dehydration sequence: 50% ethanol for 10 min, 2% uranyl acetate in 70% ethanol for 40 min, 85% ethanol for 10 min, 95% for 10 min, and 100% for 30 min. Samples were embedded in EM812 epoxy resin (Electron Microscopy Sciences) with 2,4,6-tris-(dimethylaminomethyl) phenol (DMP-30) and cured at 60 °C for 48–72 h. Samples were sectioned with a Leica UCT Ultracut ultramicrotome into 1 *μ*m sections and imaged using a JEOL JEM-1400 Flash TEM. The TEM embedding, sectioning, and imaging were performed by the Integrated Biomedical Imaging Services (IBIS) at the Boston University Medical School Campus. The Transmission Electron Microscope was funded by the NIH Award No. S10OD028571.

### H. Statistical analyses

Statistical tests were performed in JMP Pro 15 software. First, the Shapiro–Wilk test was performed on each dataset to determine if the data were normally distributed. The specific statistical tests used are specified in figure captions; generally, the Dunnett's test with control was performed for normally distributed data, and the Steel test with control was performed for non-normally distributed data. A p-value < 0.05 was considered significant. For Figs. 1, 2, and S1, at least three biological replicates were performed with at least five tissues per condition in each replicate. For Fig. 3(b), one biological replicate was performed that included three tissues per condition. For Fig. 3(d), at least two biological replicates were performed with at least ten tissues per condition in each replicate. For Fig. 4, one tissue was imaged per condition with at least ten fields of view per tissue. Values given in the text are expressed as mean ± standard error of the mean.

## SUPPLEMENTARY MATERIAL

See the supplementary material for details, which includes an explanation of how laser fluence was calculated, which includes Fig. S1: details of laser ablation system and Table S1: measured laser power and calculated laser fluence. Next are supplementary figures cited in the Results section: Fig. S2: quantification of damaged tissue area, Fig. S3: treatment of microtissues with latrunculin A, and Fig. S4: ethidium staining following gap closure.

## Data Availability

The data that support the findings of this study are available from the corresponding authors upon reasonable request.
